# Idade, Insuficiência Renal e Transfusão são Preditores de Risco de Longa Permanência Hospitalar após Cirurgia de Revascularização do Miocárdio

**DOI:** 10.36660/abc.20230769

**Published:** 2024-06-06

**Authors:** Diego Pereira Gregório de Andrade, Fabiane Letícia de Freitas, Gabrielle Barbosa Borgomoni, Maxim Goncharov, Pedro Gabriel Melo de Barros e Silva, Marcelo Arruda Nakazone, Valquiria Pelisser Campagnucci, Marcos Gradim Tiveron, Luiz Augusto Lisboa, Luís Alberto Oliveira Dallan, Fabio Biscegli Jatene, Omar Asdrúbal Vilca Mejia

**Affiliations:** 1 Instituto do Coração do Hospital das Clínicas da Faculdade de Medicina da Universidade de São Paulo São Paulo SP Brasil Instituto do Coração do Hospital das Clínicas da Faculdade de Medicina da Universidade de São Paulo, São Paulo, SP – Brasil; 2 Instituto de Pesquisa Hospital do Coração São Paulo SP Brasil Hospital do Coração - Instituto de Pesquisa, São Paulo, SP – Brasil; 3 Hospital Samaritano Paulista São Paulo SP Brasil Hospital Samaritano Paulista, São Paulo, SP – Brasil; 4 Faculdade de Medicina de São José do Rio Preto São José do Rio Preto SP Brasil Faculdade de Medicina de São José do Rio Preto, São José do Rio Preto, SP – Brasil; 5 Faculdade de Ciências Médicas da Santa Casa de São Paulo São Paulo SP Brasil Faculdade de Ciências Médicas da Santa Casa de São Paulo, São Paulo, SP – Brasil; 6 Irmandade da Santa Casa de Misericórdia de Marília Marília SP Brasil Irmandade da Santa Casa de Misericórdia de Marília, Marília, SP – Brasil

**Keywords:** Tempo de Internação, Revascularização Miocárdica, Otimização de Processos

## Abstract

**Fundamento:**

A identificação de fatores de riscos na cirurgia cardiovascular auxilia na previsibilidade resultando na otimização de desfechos e redução de custos.

**Objetivo:**

Identificação dos preditores de risco pré e intraoperatórios para internação prolongada após cirurgia de revascularização do miocárdio (CRM) no Estado de São Paulo.

**Métodos:**

Análise transversal no banco de dados REPLICCAR II, registro prospectivo, consecutivo, multicêntrico que incluiu cirurgias de revascularização miocárdica realizadas entre agosto de 2017 e julho de 2019. O desfecho principal foi o tempo de internação prolongado, definida como período de pós-operatório superior a 14 (quatorze) dias. Para a identificação dos preditores foram realizadas análises de regressão logística uni- e multivariada. Os valores de p menores de 0,05 foram considerados significativos.

**Resultados:**

A mediana de idade foi de 63 (57-70) anos e 26,55% eram do sexo feminino. Dos 3703 pacientes analisados, 228 (6,16%) apresentaram longa permanência hospitalar (LPH) após a CRM e a mediana da internação foi de 17 (16-20) dias. Foram preditores da LPH após a CRM: idade >60 anos (OR 2,05; IC95% 1,43 - 2,87; p<0,001); insuficiência renal (OR 1,73; IC95% 1,29 - 2,32; p<0,001) e transfusão de hemácias no intraoperatório (OR 1,32; IC 1,07 - 2,06; p=0,01).

**Conclusão:**

Nesta análise, a idade > 60 anos, insuficiência renal e a transfusão de hemácias no intraoperatório foram preditores independentes de LPH após a CRM. A identificação destas variáveis pode ajudar no planejamento estratégico multiprofissional visando melhoria de resultados e otimização de recursos no estado de São Paulo.

## Introdução

O tempo de internação hospitalar pós-operatória é um forte indicador de qualidade dos serviços de saúde,^1 - 3^ utilizado como parâmetro para gerenciamento de recursos na gestão pública e privada dos serviços de saúde.^3 , 4^ O Brasil, por exemplo, desde 2022 tem modelo público de pagamento baseado em métricas de qualidade e desempenho considerando o tempo de internação hospitalar pós-operatório como uma das variáveis utilizadas.^4^ Com evidências consolidadas sobre a redução dos desfechos cardiovasculares no longo prazo,^5^ a cirurgia de revascularização do miocárdio (CRM) pode contribuir para uma longa permanência hospitalar (LPH), trazendo riscos aos pacientes, impacto na qualidade de vida, aumento de custos às fontes pagadoras do sistema de saúde^1^ e, gerando interferência negativa nos indicadores de qualidade do serviço hospitalar.^6^

Nos Estados Unidos, em uma coorte multicêntrica,^7^ analisou-se a relação de tempo de internamento versus custos ao sistema de saúde, baseando-se em dados de 42.839 pacientes submetidos a CRM. Nesse estudo identificou-se que uma internação média de 5,4 dias custava US$33.275,00, enquanto uma média de internação de 13,8 dias custava US$69.122,00 às fontes pagadoras. No Brasil, segundo dados do DATASUS,^8^ entre janeiro e novembro de 2022 foram realizadas 17.931 CRM isoladas pelo Sistema Único de Saúde, custando R$180.123.355,01 em serviços hospitalares. Ainda segundo esses dados,^8^ o tempo de permanência hospitalar médio para uma CRM com uso de circulação extracorpórea com 2 ou mais enxertos, foi de 12,1 dias no período citado. Reduzir o tempo de internação hospitalar, seus custos e complicações associadas, são metas de diferentes linhas de estudos com protocolos de rápida recuperação após CRM.^9^

A literatura disponível sobre fatores que levam à LPH após CRM tem uma variedade de métodos utilizados, alguns com tamanhos de amostras limitados, diferentes critérios de alocação dos pacientes,^2 , 10 - 13^ e resultados com diferentes fatores de impactos para LPH após CRM. Características socioeconômicas, além das clínicas e hospitalares, mostraram-se influentes no tempo de internação pós-operatória,^2 , 11^ sendo necessários estudos específicos para as diferentes populações. Os estudos direcionados à população brasileira são insuficientes para gerar previsibilidade, seja por amostra limitada^13^ ou metodologias direcionadas para poucas variáveis,^14^ justificando a necessidade de dados mais robustos relacionados a este tema.

Neste âmbito, é de fundamental importância identificar e compreender os fatores que levam ao aumento do tempo de internação após CRM, visando aumentar a previsibilidade, melhorar desfechos e reduzir custos aos sistemas de saúde.^3^ Este estudo teve como objetivo identificar preditores de risco de LPH após CRM.

## Métodos

Esta é uma análise transversal do banco de dados REPLICCAR II,^15^ um registro prospectivo, observacional e multicêntrico que incluiu cirurgias de revascularização miocárdica realizadas em 5 hospitais do estado de São Paulo, entre agosto de 2017 e julho de 2019.

O banco de dados REPLICCAR II^15^ contém pacientes com idade ≥ 18 anos submetidos à CRM primária e isolada de forma eletiva, e urgência. A plataforma para coleta de dados foi criada no REDCap ( http://www.project-redcap.org ) especialmente para o projeto, na qual foi coletado de forma online por profissionais graduados e treinados. O banco de dados contém as mesmas variáveis e definições da versão 2.9 do sistema de coletas do STS ( *Society of Thoracic Surgeons* ).^11^

### Qualidade de dados

No total, o banco de dados REPLICCAR II^15^ inclui 4049 pacientes, 346 foram excluídos desta análise devido à ausência de dados referentes à permanência hospitalar.

### Definição das variáveis

A LPH após CRM foi definida como internação hospitalar maior que 14 dias após a realização de CRM, seguindo a definição utilizada pelo banco de dados do STS.^11^ Transfusão intraoperatória referiu-se a infusão de concentrado de hemácias durante o procedimento cirúrgico. Para cálculo do Clearance de creatinina foi utilizada a equação de Crockoft-Gault.^16^ Cirurgias de emergências foram excluídas desta análise por considerar que fatores relacionados com a gravidade dos pacientes poderiam enviesar a identificação de preditores passíveis de gerenciamento.

### Análise estatística

O software R versão 4.0.2 foi utilizado para a realização de todas as análises realizadas neste estudo.

Na análise descritiva, as variáveis contínuas apresentaram assimetria e, por isso, foram descritas através de mediana e intervalo interquartil, enquanto as variáveis categóricas foram expressas em termos de frequências e porcentagens.

As variáveis independentes categóricas foram analisadas por meio da comparação de proporções com os testes qui-quadrado ou exato de Fisher, conforme apropriado. O teste de normalidade foi realizado fazendo uso do Shapiro-Wilk e o teste de homogeneidade de amostras por intermédio do teste de Levene. O teste de Mann-Whitney foi utilizado para análise das variáveis contínuas devido à distribuição dos dados.

As variáveis de predição (pré e intraoperatórias) foram analisadas por meio da regressão logística univariada, e as variáveis com valor de p menor que 0,05 foram submetidas consecutivamente a um modelo de regressão logística multivariada para avaliar o impacto independente dos preditores na permanência prolongada pós-operatória.

Foram expressas a razão de chances ( *odds ratio* ) e o intervalo de confiança de 95%. Os valores de p menores de 0,05 foram considerados significativos.

### Ética e termo de consentimento

Esta é uma análise do registro REPLICCAR II,^15^ aprovado pela Comissão de Ética com o número de parecer 5.603.742, sob o número de registro CAAE: 66919417.6.1001.0068 e SDC 4506/17/006 aprovado em 10-04-2017. O consentimento livre e esclarecido foi dispensado na coleta de dados devido à metodologia do desenho de pesquisa, aplicada ao projeto inicial.

## Resultados

Foram avaliados 3703 pacientes submetidos à CRM. Destes, 228 (6,16%) apresentaram internação prolongada no pós-operatório com a mediana de 17 (16-20) dias.

Na Tabela 1 , são apresentadas as características dos dois grupos avaliados. Quanto à idade, o grupo que teve LPH após a CRM demonstrou uma mediana mais elevada. No que diz respeito ao gênero, a prevalência feminina foi mais acentuada nesse grupo, e o índice de massa corpórea ≥ 30, bem como pacientes com admissão em urgência, foi mais comum no mesmo.

**Tabela 1:** Características dos pacientes com longa permanência hospitalar após CRM, REPLICCAR II, São Paulo, 2022

		Tempo de permanência após CRM					
Características		≤ 14 dias (n=3475)		> 14 dias (n=228)			
		n	%	n	%	IC 95%	Valor de p
Idade (anos) *		63 (57-70) *		67 (62-72) *		62,76 a 63,35	< 0,001 §
Sexo (feminino)		908	26,13	75	32,89	0,25 a 0,28	0,02 ‡
Urgência (admissão)		1505	43,31	118	51,75	0,42 a 0,45	0,01 §
Índice de massa corporal (kg/m^2^)							
	< 18,5	15	0,44	0	-	0,002 a 0,007	0,01 †
	18,5-24,9	1064	30,87	67	29,78	0,31 a 0,34	
	25-29,9	1513	43,89	42	18,67	0,31 a 0,34	
	≥ 30	855	24,80	116	51,56	0,27 a 0,29	
Infarto prévio do miocárdio		1821	52,4	131	57,46	0,51 a 0,54	0,13 ‡
Hipertensão arterial sistêmica		3072	88,4	198	88,64	0,87 a 0,89	0,47 ‡
Diabetes mellitus		1690	45,13	111	48,68	0,44 a 0,47	0,98 ‡
Doença cerebrovascular 1		314	9,04	31	13,6	0,08 a 0,10	0,02 ‡
Fibrilação atrial		50	1,44	5	2,19	0,01 a 0,02	0,39 †
Fração de ejeção (<30%)		49	1,41	8	3,51	0,01 a 0,02	0,02 †
Insuficiência renal 2		955	27,48	112	49,12	0,27 a 0,30	< 0,001 ‡
Classificação de angina CCS							
	IV	325	9,35	31	13,6	0,08 a 0,10	0,03 ‡
Classificação NYHA							
	I e II	3051	87,8	185	81,14	0,86 a 0,88	<0,001 ‡
	III e IV	424	12,2	43	18,86	0,11 a 0,13	
Anemia 3		1263	36,35	112	49,12	0,35 a 0,38	< 0,001 ‡
Transfusão de hemácias no intraoperatório		560	16,12	66	28,95	0,16 a 0,18	< 0,001 ‡
Utilização de circulação extracorpórea		3163	91,02	201	88,16	0,89 a 0,91	0,14 ‡
STS score (permanência prolongada) *		1,66 (1,10 - 2,61) *		2,11 (1,43 - 3,31) *		2,14 a 2,26	< 0,001 §
Tempo de permanência após CRM *		7 (5-8) *		17 (16-20) *		7.52 a 7.75	< 0,001 §
STS score (mortalidade) *		0,62 (0,41- 0,99) *		0,8 (0,53 - 1,21) *		0,82 a 0,87	< 0,001 §
Óbito		57	1,64	10	4,38	0,01 a 0,02	0,007 †

1 Doença cerebrovascular: Acidente vascular cerebral, ataque isquêmico transitório ou estenose das carótidas

2 Considerado Clearance de creatinina < 60 ml/min/1,73m^2^

3 Anemia: Hemoglobina <11,9mg/dL para mulheres e <13,6mg/dL para homens;^17^ NYHA: New York Heart Association; CSC: Canadian Cardiovascular Society; STS: Society of Thoracic Surgeons; CRM: cirurgia de revascularização do miocárdio.

* Mediana e intervalo interquartil

† exato de Fisher

‡ qui-quadrado

§ Mann Whitney; IC 95% (médias e proporções).

Foi observada uma maior incidência de doença cerebrovascular prévia no grupo LPH em comparação com o grupo que passou por internação pós-operatória de até 14 dias. Entre os pacientes com LPH, uma proporção significativamente maior apresentou fração de ejeção cardíaca inferior a 30%, enquanto no grupo com internação pós-operatória de até 14 dias, essa incidência foi menor. No que diz respeito aos pacientes com insuficiência renal, a prevalência de Clearance de creatinina inferior a 60 ml/min/1,73m^2^ foi significativamente maior no grupo submetido à LPH. Além disso, no grupo com LPH, houve uma maior incidência de pacientes classificados com angina Canadian Cardiovascular Society (CSS)^18^ IV e uma presença mais significativa de pacientes nas classes III e IV na classificação New York Heart Association (NYHA)^19^ em comparação com o grupo com internação pós-operatória de até 14 dias.

No grupo com LPH após a CRM, detectou-se uma maior incidência de pacientes com anemia pré-operatória em comparação com o grupo com internação pós-operatória de até 14 dias. Os pacientes que necessitaram de transfusão de hemácias no intraoperatório foram mais prevalentes no grupo com LPH após CRM. O risco de LPH estimado pelo STS foi mais alto nos pacientes que tiveram internação prolongada em comparação com o grupo com internação pós-operatória de até 14 dias. Da mesma forma, o risco de mortalidade estimado pelo STS foi maior nos pacientes que tiveram LPH após CRM. Infarto prévio do miocárdio, hipertensão arterial sistêmica, diabetes mellitus, fibrilação atrial e utilização de circulação extracorpórea não apresentaram significância estatística entre esses dois grupos. Após a regressão logística univariada ( Tabela 2 ), dez variáveis apresentaram relação com LPH após CRM e foram, em seguida, para a regressão logística multivariada.

**Tabela 2:** Regressão logística univariada com preditores de longa permanência hospitalar após CRM. REPLICCAR II, São Paulo, 2022

Variável		OR	IC 95 %	Valor de p
Idade (>60 anos)		2,62	1,89 a 3,65	< 0,001
Sexo (feminino)		1,38	1,04 a 1,84	0,02
Urgência (admissão)		1,40	1,07 a 1,83	0,01
Doença cerebrovascular 1		1,58	1,07 a 2,35	0,022
Fração de ejeção (< 30%)		3,02	1,3 a 6,83	0,03
Insuficiência renal 2		2,55	1,00 a 3,43	< 0,001
Classificação de angina CCS				
	IV	1,52	1,03 a 2,26	0,04
Classificação NYHA				
	III e IV	1,67	1,18 a 2,36	0,004
Anemia 3		1,69	1,29 a 2,21	< 0,001
Transfusão de hemácias no intraoperatório		2,12	1,57 a 2,86	< 0,001

1 Doença cerebrovascular: Acidente vascular cerebral, ataque isquêmico transitório ou estenose das carótidas

2 Considerado Clearance de creatinina < 60 ml/min/1,73m^2^

3 Anemia: Hemoglobina < 11,9mg/dL para mulheres e < 13,6mg/dL para homens;17 CCS: Canadian Cardiovascular Society; NYHA: New York Heart Association; OR: Oddsratio.

Das variáveis incluídas na regressão logística multivariada ( Tabela 3 ), três variáveis mostraram associação com a internação pós-operatória prolongada: idade, insuficiência renal e transfusão de hemácias no intraoperatório ( Figura Central ).

**Figure:**
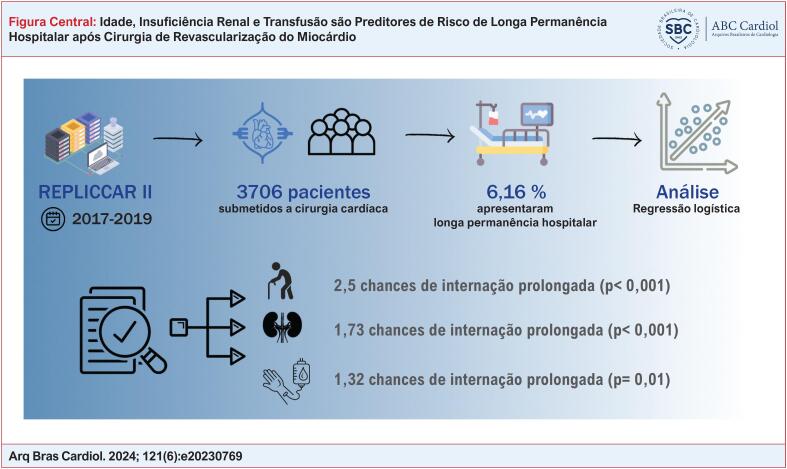


**Tabela 3:** Regressão logística multivariada com preditores de longa permanência hospitalar após CRM. REPLICCAR II, São Paulo, 2022

Variável	OR	IC 95 %	Valor de p
Idade (>60 anos)	2,05	1,43 a 2,87	< 0,001
Insuficiência renal 1	1,73	1,29 a 2,32	< 0,001
Transfusão de hemácias no intraoperatório	1,32	1,07 a 2,06	0,01

1 Considerado Clearance de creatinina < 60 ml/min/1,73m^2^.

CRM de urgência, assim como sexo, histórico de doença cerebrovascular, anemia pré-operatória, fração de ejeção < 30%, presença de angina CCS IV, bem como classe funcional NYHA III e IV, não representaram significância estatística no tempo de permanência pós-operatória dos pacientes.

## Discussão

Na amostra deste estudo 6,15% (n=228) dos pacientes apresentaram internação prolongada, achado compatível com estudos prévios,^2 , 10^ mas ainda acima das médias encontradas nos estudos de rápida recuperação após cirurgia cardíaca.^9^ Os preditores de LPH após CRM encontrados nesta análise (idade >60 anos, insuficiência renal, e transfusão de hemácias no intraoperatório) diferem da literatura relativa à população brasileira, com objetivo semelhante,^13^ porém estão alinhados com dados da literatura mundial elaborados com análises estatísticas diferentes.^12^ Um estudo norte-americano com 2121 pacientes submetidos a CRM^12^ em um único centro, analisando 116 variáveis, com 2 (duas) diferentes técnicas de análise de dados por inteligência artificial, apontou como 4 principais fatores de impacto para LPH após CRM: tempo de intubação, valor de creatinina pré-operatório, idade e número de transfusões intraoperatórias.

O impacto da idade >60 anos no aumento do tempo de permanência hospitalar, encontrado neste estudo, está alinhado com dados de trabalhos prévios.^2 , 10^ Em um estudo com 649 pacientes submetidos a CRM,^2^ utilizou-se testes paramétricos univariados e modelo de regressão linear múltipla, para identificar preditores de LPH após CRM, apontando a idade como variável independente. Outro estudo com 1426 pacientes^10^ do banco de dados STS^11^ utilizou modelo baseado em inteligência artificial, conhecido como algoritmo genético, que propôs 23 fatores pré e intraoperatórios relacionados com aumento da permanência hospitalar após CRM, sendo a idade um dos 3 principais fatores encontrados. Os modelos de predição de risco cirúrgico do STS^11^ e o Euroscore II^20^ colocam a idade como um fator de risco isolado para aumento de morbimortalidade após cirurgia cardíaca. As variações orgânicas relacionadas à idade e ao aumento de comorbidades nas populações idosas são fatores sugeridos para o aumento de complicações pós-operatórias e tempo de internação hospitalar após CRM.^21^

A disfunção renal pré-operatória é um conhecido fator de piora de desfechos a curto e longo prazo após CRM.^22 , 23^ Apesar do amplo uso da creatinina como biomarcador de função renal, seus níveis séricos normais ≤ 1,3mg/dL^23^ podem mascarar uma disfunção renal existente, identificada pelo Clearance de creatinina < 60ml/min/1,73m^2^, conhecida como disfunção renal oculta.^22 , 24^ Esta por sua vez é um fator de risco independente para mortalidade,^23 , 24^ disfunção renal pós-operatória,^23 , 24^ hemodiálise,^23 , 24^ acidente vascular encefálico,^23^ e internação hospitalar > 7 dias,^23 , 24^ após CRM. Estas condições podem aumentar o tempo de internação hospitalar após a cirurgia.^22 - 24^

A associação entre transfusão de hemácias e eventos adversos no pós-operatório de CRM é consistentemente descrita na literatura.^25 - 28^ A relação entre aumento do tempo de internamento hospitalar e hemotransfusão, encontrada neste estudo, é corroborada com a literatura existente.^26 - 29^ Há diversas razões para esta associação tais quais: infecções, arritmias, insuficiência renal aguda, acidente vascular encefálico.^27 - 29^ Em uma coorte prospectiva, multicêntrica nos Estados Unidos, a transfusão de concentrado de hemácias foi identificada como fator de risco independente para aumento de tempo de permanência em unidade de terapia intensiva e tempo de internação hospitalar.^25^ Este resultado foi semelhante a um estudo prospectivo observacional que encontrou as mesmas associações independentemente do valor de hemoglobina pré transfusional.^26^

Com base nos dados deste estudo e de estudos prévios, entende-se que intervenções realizadas nas variáveis de impacto tem potencial de reduzir o risco de internação pós-operatória prolongada, tais quais: medidas preventivas e terapêuticas específicas para pacientes > 60 anos; diagnóstico de disfunção renal no pré-operatório e medidas de nefro proteção perioperatórias; técnicas de diminuição de transfusão de hemácias no intraoperatório.

### Limitações

Neste estudo foi utilizada a técnica de regressão logística para identificação dos preditores de LPH após CRM. Apesar da eficiência deste tipo de análise, atualmente estão sendo utilizadas técnicas com inteligência artificial que se propõem trabalhar grandes bancos de dados com tal objetivo e menor probabilidade de erro.

A falta de dados referente ao tempo de permanência pós-operatória levou à exclusão de pacientes para esta análise limitando assim o tamanho da amostra para a análise. Apesar disso, a análise realizada teve um número maior de eventos que o necessário para estas análises, além de ser a maior amostra de estudos prévios na população brasileira, sendo compatível com a literatura mundial.

Nesta análise não foi realizada a construção de um escore de risco capaz de predizer o risco de LPH após CRM, no entanto estes dados podem servir de parâmetro para a construção de tal modelo em estudos futuros.

## Conclusão

Pacientes com mais de 60 anos com insuficiência renal e transfusão de hemácias no intraoperatório foram preditores independentes de LPH após a CRM. Estas variáveis devem ser validadas em outras populações para confirmar sua acurácia e podem ser levadas em consideração no planejamento estratégico multiprofissional visando otimização de resultados e recursos do sistema de saúde.
