# Supplementary material for: Idade, Insuficiência Renal e Transfusão são Preditores de Risco de Longa Permanência Hospitalar após Cirurgia de Revascularização do Miocárdio
Source: Arq Bras Cardiol. 2024 Jun 6;121(6):e20230769. [Article in Portuguese] doi: 10.36660/abc.20230769 (PMC12092024; doi:10.36660/abc.20230769)
Supplement: Translation

# Translation: Age, Renal Failure and Transfusion are Risk Predictors of Prolonged Hospital Stay after Coronary Artery Bypass Grafting Surgery

**DOI:** 10.36660/abc.20230769i

**Authors:** Diego Pereira Gregório de Andrade, Fabiane Letícia de Freitas, Gabrielle Barbosa Borgomoni, Maxim Goncharov, Pedro Gabriel Melo de Barros e Silva, Marcelo Arruda Nakazone, Valquiria Pelisser Campagnucci, Marcos Gradim Tiveron, Luiz Augusto Lisboa, Luís Alberto Oliveira Dallan, Fabio Biscegli Jatene, Omar Asdrúbal Vilca Mejia

**Affiliations:** 1 São Paulo SP Brazil Instituto do Coração do Hospital das Clínicas da Faculdade de Medicina da Universidade de São Paulo, São Paulo, SP – Brazil; 2 São Paulo SP Brazil Hospital do Coração - Instituto de Pesquisa, São Paulo, SP – Brazil; 3 São Paulo SP Brazil Hospital Samaritano Paulista, São Paulo, SP – Brazil; 4 São José do Rio Preto SP Brazil Faculdade de Medicina de São José do Rio Preto, São José do Rio Preto, SP – Brazil; 5 São Paulo SP Brazil Faculdade de Ciências Médicas da Santa Casa de São Paulo, São Paulo, SP – Brazil; 6 Marília SP Brazil Irmandade da Santa Casa de Misericórdia de Marília, Marília, SP – Brazil

**Keywords:** Length of Stay, Myocardial Revascularization, Process Optimization

## Abstract

**Background:**

Identifying risk factors in cardiovascular surgery assists in predictability, resulting in optimization of outcomes and cost reduction.

**Objective:**

This study aimed to identify preoperative and intraoperative risk predictors for prolonged hospitalization after coronary artery bypass grafting (CABG) surgery in the state of São Paulo, Brazil.

**Methods:**

A cross-sectional analysis using data from the REPLICCAR II database, a prospective, consecutive, multicenter registry that included CABG surgeries performed between August 2017 and July 2019. The primary outcome was a prolonged hospital stay (PHS), defined as a postoperative period exceeding 14 days. Univariate and multivariate logistic regression analyses were performed to identify the predictors with significance set at p <0.05.

**Results:**

The median age was 63 (57-70) years and 26.55% of patients were female. Among the 3703 patients analyzed, 228 (6.16%) had a PHS after CABG, with a median hospital stay of 17 (16-20) days. Predictors of PHS after CABG included age >60 years (OR 2.05; 95% CI 1.43-2.87; p<0.001); renal failure (OR 1.73; 95% CI 1.29-2.32; p <0.001) and intraoperative red blood cell transfusion (OR 1.32; 95% CI 1.07-2.06; p=0.01).

**Conclusion:**

Age >60 years, renal failure, and intraoperative red blood cell transfusion were independent predictors of PHS after CABG. The identification of these variables can help in multiprofessional strategic planning aimed to enhance results and resource utilization in the state of São Paulo.

## Introduction

The length of postoperative hospital stay is a robust measure of healthcare quality,^1 - 3^guiding resource management in both public and private health management sectors.^3 , 4^Brazil, for instance, has adopted a public payment model since 2022 that incorporates quality and performance metrics, considering the length of postoperative hospital stay as one of the variables used.^4^With consolidated evidence on the reduction of cardiovascular outcomes in the long term,^5^coronary artery bypass grafting (CABG) surgery can contribute to prolonged hospital stay (PHS), bringing risks to patients, impacting quality of life, increasing healthcare costs,^1^and adversely affecting hospital service quality indicators.^6^

In a multicenter cohort study conducted in the United States,^7^the correlation between hospital length of stay and costs to the healthcare system was analyzed, based on data from 42,839 patients who underwent CABG. The findings revealed that an average hospitalization of 5.4 days incurred a cost of US$33,275.00, whereas an average stay of 13.8 days amounted to US$69,122.00 for healthcare payers. In Brazil, according to data from DATASUS,^8^between January and November 2022, a total of 17,931 isolated CABG surgeries were carried out by the Brazilian Unified Health System, costing R$180,123,355.01. Moreover, during this period,^8^the average hospital stay for a CABG using cardiopulmonary bypass with two or more grafts was 12.1 days. Various research endeavors aiming to reduce hospital stays, associated costs, and complications following CABG are underway, focusing on the implementation of rapid recovery protocols.^9^

The existing literature on factors leading to PHS following CABG varies in methodologies, with some studies utilizing limited sample sizes and different patient allocation criteria,^2 , 10 - 13^and results with different impact factors for PHS after CABG. Socioeconomic characteristics, alongside clinical and hospital-related characteristics, have been shown to influence postoperative hospitalization duration,^2 , 11^requiring specific studies for different populations. However, studies focusing on the Brazilian population remain insufficient in providing predictive insights, either by a limited sample^13^or methodologies focused on a narrow set of variables.^14^Hence, there is a need for more robust data related to this theme.

In this context, it becomes imperative to identify and understand the factors that lead to prolonged hospitalization after CABG, aiming to increase predictability, improve outcomes, and reduce costs to healthcare system.^3^This study aimed to identify risk predictors of PHS after CABG surgery.

## Methods

This study comprises a cross-sectional analysis of the REPLICCAR II database,^15^a prospective, observational, and multicenter registry encompassing CABG surgeries performed at five hospitals in the state of São Paulo between August 2017 and July 2019.

The REPLICCAR II database^15^comprises patients aged ≥ 18 years who underwent elective and urgent CABG surgery. Data collection utilized the REDCap platform ( http://www.project-redcap.org ), especially for the project, and was collected online by qualified and trained professionals. The database contains the same variables and definitions as version 2.9 of the Society of Thoracic Surgeons (STS) data collection system.^11^

### Data quality

The REPLICCAR II^15^database includes a total of 4049 patients, however, 346 were excluded from this analysis due to the missing data regarding hospital stay.

### Variable definitions

Long hospital stay after CABG was defined as hospitalization exceeding 14 days post-surgery, following the definition used by the STS database.^11^Intraoperative transfusion was characterized by the infusion of packed red blood cells during the surgical procedure. Creatinine clearance was calculated using the Crockoft-Gault equation.^16^Emergency surgeries were excluded from this analysis to mitigate potential bias in identifying manageable predictors, as factors related to patient severity could confound the results.

### Statistical analysis

All analyses in this study were conducted using R software version 4.0.2.

In the descriptive analysis, continuous variables presented asymmetry and, therefore, were described through median and interquartile ranges. Categorical variables were expressed as frequencies and percentages.

Categorical independent variables were analyzed by comparing proportions using either the chi-square or Fisher's exact tests, as appropriate. Normality and sample homogeneity tests were performed using the Shapiro-Wilk and Levene tests, respectively. Continuous variables were analyzed using the Mann-Whitney test due to data distribution.

Prediction variables, both preoperative and intraoperative, were analyzed using univariate logistic regression. Variables with a p-value <0.05 were consecutively submitted to a multivariate logistic regression model to assess their independent impact on postoperative length of stay.

Odds ratios and their corresponding 95% confidence intervals were expressed. A significance level of p <0.05 was utilized.

### Ethics and informed consent

This analysis utilized data from the REPLICCAR II^15^registry, which received approval from the Ethics Committee under opinion number 5.603.742, CAAE registration number: 66919417.6.1001.0068 and SDC 4506/17/006 approved on 04/10/2017. Informed consent for data collection was waived due to the research design employed in the initial project.

## Results

A total of 3703 patients who underwent CABG were evaluated. Among them, 228 (6.16%) had prolonged postoperative hospitalization, with a median duration of 17 (16-20) days.

Table 1 shows the characteristics of the two evaluated groups. The group experiencing PHS after CABG demonstrated a higher median age. Additionally, there was a higher prevalence of females in this group, and a higher proportion of patients with a body mass index ≥30, as well as emergency admissions.

**Table 1:** Characteristics of patients with prolonged hospital stay after CABG, REPLICCAR II, São Paulo, 2022

		Length of stay after CABG					
Characteristics		≤ 14 days (n=3475)		> 14 days (n=228)			
		n	%	n	%	95% CI	p-value
Age (years)*		63 (57-70) *		67 (62-72) *		62.76 a 63.35	< 0.001 §
Gender (female)		908	26.13	75	32.89	0.25 a 0.28	0.02 ‡
Urgency (admission)		1505	43.31	118	51.75	0.42 a 0.45	0.01 §
Body mass index (kg/m^2^)							
	<18.5	15	0.44	0	-	0.002 a 0.007	0.01 †
	18.5-24.9	1064	30.87	67	29.78	0.31 a 0.34	
	25-29.9	1513	43.89	42	18.67	0.31 a 0.34	
	≥ 30	855	24.80	116	51.56	0.27 a 0.29	
Previous myocardial infarction		1821	52.4	131	57.46	0.51 a 0.54	0.13 ‡
Systemic arterial hypertension		3072	88.4	198	88.64	0.87 a 0.89	0.47 ‡
Diabetes mellitus		1690	45.13	111	48.68	0.44 a 0.47	0.98 ‡
Cerebrovascular disease 1		314	9.04	31	13.6	0.08 a 0.10	0.02 ‡
Atrial fibrillation		50	1.44	5	2.19	0.01 a 0.02	0.39 †
Ejection fraction (<30%)		49	1.41	8	3.51	0.01 a 0.02	0.02 †
Renal failure 2		955	27.48	112	49.12	0.27 a 0.30	< 0.001 ‡
CCS angina class							
	IV	325	9.35	31	13.6	0.08 a 0.10	0.03 ‡
NYHA functional class							
	I and II	3051	87.8	185	81.14	0.86 a 0.88	<0.001 ‡
	III and IV	424	12.2	43	18.86	0.11 a 0.13	
Anemia 3		1263	36.35	112	49.12	0.35 a 0.38	< 0.001 ‡
Intraoperative red blood cell transfusion		560	16.12	66	28.95	0.16 a 0.18	< 0.001 ‡
Use of cardiopulmonary bypass		3163	91.02	201	88.16	0.89 a 0.91	0.14 ‡
STS score (prolonged stay)*		1.66 (1.10 - 2.61) *		2.11 (1.43 - 3.31) *		2.14 a 2.26	< 0.001 §
Length of stay after CABG*		7 (5-8) *		17 (16-20) *		7.52 a 7.75	< 0.001 §
STS score (mortality)*		0.62 (0.41- 0.99) *		0.8 (0.53 - 1.21) *		0.82 a 0.87	< 0.001 §
Death		57	1.64	10	4.38	0.01 a 0.02	0.007 †

1 Cerebrovascular disease: stroke, transient ischemic attack, or carotid stenosis

2 Creatinine clearance <60 ml/min/1.73 m^2^

3 Anemia: Hemoglobin <11.9 mg/dL for women and <13.6 mg/dL for men;^17^CCS: Canadian Cardiovascular Society; NYHA: New York Heart Association; OR: odds ratio.

The PHS group exhibited a higher incidence of previous cerebrovascular disease compared to the group with a postoperative hospital stay of up to 14 days. Among patients with PHS, a significantly higher proportion had a cardiac ejection fraction <30%, while this incidence was lower in the group with postoperative hospitalization of up to 14 days. Regarding patients with renal failure, the prevalence of creatinine clearance <60 ml/min/1.73 m^2^was significantly higher in the PHS group. In addition, among patients in the PHS group, there was a higher incidence of those classified with angina class IV according to the Canadian Cardiovascular Society (CCS)^18^and a more significant presence of patients in functional classes III and IV according to New York Heart Association (NYHA),^19^compared to the group with postoperative hospitalization of up to 14 days.

In the group experiencing PHS following CABG, there was a higher incidence of patients with preoperative anemia compared to those with postoperative hospitalization of up to 14 days. Patients requiring intraoperative red blood cell transfusion were more prevalent in the PHS group. The risk of PHS estimated by STS was higher in patients with prolonged hospitalization compared to the group with postoperative hospitalization of up to 14 days. Similarly, the mortality risk estimated by STS was higher in patients who had PHS after CABG. Previous myocardial infarction, systemic arterial hypertension, diabetes mellitus, atrial fibrillation, and the use of cardiopulmonary bypass did not show statistically significant differences between these two groups. Following univariate logistic regression ( Table 2 ), ten variables were associated with PHS after CABG and were subsequently included in multivariate logistic regression analysis.

**Table 2:** Univariate logistic regression with predictors of prolonged hospital stay after CABG. REPLICCAR II, São Paulo, 2022

Variable		OR	95 % CI	p-value
Age >60 years		2.62	1.89 a 3.65	< 0.001
Gender (female)		1.38	1.04 a 1.84	0.02
Urgency (admission)		1.40	1.07 a 1.83	0.01
Cerebrovascular disease 1		1.58	1.07 a 2.35	0.022
Ejection fraction (<30%)		3.02	1.3 a 6.83	0.03
Renal failure 2		2.55	1.00 a 3.43	< 0.001
CCS angina class				
	IV	1.52	1.03 a 2.26	0.04
NYHA functional class				
	III and IV	1.67	1.18 a 2.36	0.004
Anemia 3		1.69	1.29 a 2.21	< 0.001
Intraoperative red blood cell transfusion		2.12	1.57 a 2.86	< 0.001

1 Cerebrovascular disease: stroke, transient ischemic attack, or carotid stenosis

2 Creatinine clearance <60 ml/min/1.73 m^2^

3 Hemoglobin <11.9 mg/dLFor women and <13.6 mg/dL for men;^17^CCS: Canadian Cardiovascular Society; NYHA: New York Heart Association; OR: odds ratio.

Among the variables included in the multivariate logistic regression ( Table 3 ), three variables demonstrated an association with prolonged postoperative hospitalization: age, renal failure, and intraoperative red blood cell transfusion ( central figure ).

**Table 3:** Univariate logistic regression with predictors of prolonged hospital stay after CABG. REPLICCAR II, São Paulo, 2022

Variable	OR	95 % CI	p-value
Age >60 years	2.05	1.43 a 2.87	< 0.001
Renal failure 1	1.73	1.29 a 2.32	< 0.001
Intraoperative red blood cell transfusion	1.32	1.07 a 2.06	0.01

1 Creatinine clearance < 60 ml/min/1.73m^2^.

Urgent CABG, as well as gender, history of cerebrovascular disease, preoperative anemia, ejection fraction < 30%, presence of angina CCS class IV, as well as NYHA functional class III and IV, did not show statistical significance in the length of postoperative stay.

## Discussion

In the sample of this study, 6.15% (n=228) of patients experienced prolonged hospitalization, a finding compatible with previous studies,^2 , 10^albeit slightly higher than averages found in studies investigating rapid recovery after cardiac surgery.^9^The predictors of PHS after CABG found in this analysis (age >60 years, renal failure, and intraoperative red blood cell transfusion) differ from the literature related to the Brazilian population, with a similar objective,^13^but are in line with data from the world literature elaborated with diverse statistical analyzes.^12^A North American study with 2121 patients who underwent CABG^12^in a single center, which analyzed 116 variables with two different artificial intelligence techniques for data analysis, identified four main impact factors for PHS after CABG: intubation time, preoperative creatinine value, age, and number of intraoperative transfusions.

The observed impact of age >60 years on the increase in length of hospital stay, as evidenced in this study, is in line with data from previous studies.^2 , 10^In a study with 649 patients undergoing CABG,^2^both univariate parametric tests and a multiple linear regression model were used to identify predictors of PHS after CABG, with age as an independent variable. Another study with 1426 patients^10^from the STS^11^database used an artificial intelligence-based model, known as a genetic algorithm, which identified 23 pre- and intraoperative factors related to increased hospital stay after CABG, with age being one of the three main factors found. Both the STS^11^and EuroSCORE II^20^surgical risk prediction models emphasize age as an isolated risk factor for increased morbidity and mortality after cardiac surgery. Age-related physiological variations and increased comorbidities in elderly populations are suggested factors for increased postoperative complications and PHS after CABG.^21^

Preoperative renal dysfunction is recognized as a significant factor contributing to adverse short- and long-term outcomes after CABG.^22 , 23^Despite the widespread use of creatinine as a biomarker of renal function, normal serum levels ≤1.3 mg/dL^23^may mask underlying renal dysfunction, characterized by creatinine clearance <60 ml/min/1.73 m^2^, known as hidden renal dysfunction.^22 , 24^This hidden dysfunction serves as an independent risk factor for mortality,^23 , 24^postoperative renal dysfunction,^23 , 24^need for hemodialysis,^23 , 24^cerebrovascular accident,^23^and hospitalization >7 days^23 , 24^after CABG. These conditions may contribute to PHS after surgery.^22 - 24^

The association between red blood cell transfusion and adverse events in the postoperative period of CABG is consistently described in the literature.^25 - 28^The link between PHS and blood transfusion, as observed in this study, is supported by the existing literature.^26 - 29^Several factors contribute to this association, such as infections, arrhythmias, acute renal failure, and cerebrovascular accident.^27 - 29^In a prospective, multicenter cohort study conducted in the United States, transfusion of packed red blood cells was identified as an independent risk factor for PHS in the intensive care unit and overall length of hospital stay.^25^This finding aligns with a prospective observational study that found the same associations regardless of the pre-transfusion hemoglobin value.^26^

Based on the data from this study and previous studies, it is understood that interventions targeting the impact variables have the potential to reduce the risk of prolonged postoperative hospitalization, such as preventive and therapeutic measures specific for patients >60 years; diagnosis of preoperative renal dysfunction and perioperative nephroprotection measures; techniques to reduce intraoperative red blood cell transfusion.

### Limitations

In this study, logistic regression was used to identify predictors of PHS following CABG. While logistic regression is efficient for such analyses, artificial intelligence techniques are increasingly employed, particularly for handling large databases with similar objectives, offering potentially reduced error probabilities.

The lack of data regarding postoperative length of stay led to the exclusion of certain patients from this analysis, thus limiting the sample size. Nonetheless, the analysis performed had a greater number of events, surpassing the requirements for such analyses. In addition, this study represents the largest sample among previous studies conducted in the Brazilian population, aligning well with the global literature.

In this analysis, the construction of a risk score capable of predicting the risk of PHS after CABG was not performed. However, these data can serve as a parameter for the development of such a model in future studies.

## Conclusion

Patients aged over 60 years, along with renal failure and intraoperative red blood cell transfusion, were independent predictors of PHS after CABG. These variables should be validated in other populations to confirm their accuracy and can be considered in multiprofessional strategic planning aimed at optimizing healthcare system results and resource utilization.
